# MitoGEx: An Integrated Platform for Streamlined Human Mitochondrial Genome Analysis

**DOI:** 10.3390/genes17030338

**Published:** 2026-03-18

**Authors:** Kongpop Jeenkeawpiam, Pemikar Srifa, Natakorn Nokchan, Natthapon Khongcharoen, Anas Binkasem, Surasak Sangkhathat

**Affiliations:** 1 Department of Biomedical Sciences and Biomedical Engineering, Faculty of Medicine, Prince of Songkla University, Songkhla 90110, Thailand; kongpop.je@gmail.com (K.J.); natthapon.bond@gmail.com (N.K.); anasbin1997@gmail.com (A.B.); 2 Translational Medicine Research Center, Faculty of Medicine, Prince of Songkla University, Songkhla 90110, Thailand; nat.nokchan@gmail.com; 3 Department of Surgery, Faculty of Medicine Siriraj Hospital, Mahidol University, Bangkok 10700, Thailand

**Keywords:** mitochondrial DNA, mitochondrial diseases, mitochondrial genome analysis, computational tools, bioinformatics, MitoGEx

## Abstract

**Background/Objectives**: Mitochondrial DNA (mtDNA) is an important resource for understanding human ancestry, population diversity, and the molecular mechanisms of mitochondrial diseases. However, analyzing mtDNA thoroughly often requires advanced bioinformatics skills and command-line knowledge. To address this challenge, we created Mitochondrial Genome Explorer (MitoGEx), a user-friendly computational pipeline optimized for human mtDNA analysis that combines multiple mtDNA analysis modules within a single graphical user interface. **Methods**: The platform simplifies key analytical steps, such as quality control, sequence alignment, alignment quality assessment, variant detection, haplogroup classification, and phylogenetic reconstruction. Users can choose between Quick and Advanced modes, which offer default settings or customizable options based on their analysis needs. To demonstrate its effectiveness, we analyzed 15 whole-exome sequencing (WES) samples from Songklanagarind Hospital using MitoGEx. **Results**: The sequencing data were of high quality, with over 92 percent of bases scoring above a Phred score and consistent GC content across all samples. Variant detection using the GATK mitochondrial pipeline and annotation with ANNOVAR and the MitImpact database revealed multiple high-confidence variants. Haplogroup classification with Haplogrep 3 and phylogenetic analysis with IQ-TREE 2 confirmed diverse maternal lineages within the cohort. **Conclusions**: Taken together, MitoGEx facilitates mitochondrial genome analysis in a reproducible and accessible manner for both research and clinical bioinformatics applications. The analytical results produced by MitoGEx are concordant with those obtained using standalone bioinformatic tools, demonstrating analytical correctness. By integrating all analysis steps into a single automated workflow, MitoGEx reduces execution time and limits human error inherent to manual, multi-step pipelines.

## 1. Introduction

High-throughput sequencing technologies and bioinformatics tools have enhanced researchers’ ability to study mitochondrial DNA (mtDNA), which is extracted from mitochondria, the cell’s “powerhouses” [1]. Mitochondria produce ATP through oxidative phosphorylation and play key roles in various cellular processes, including apoptosis, calcium homeostasis, and metabolic regulation [2]. Unlike nuclear DNA, mtDNA is a small, circular, double-stranded molecule inherited exclusively through the maternal line [3]. It encodes essential proteins required for oxidative phosphorylation and has a high mutation rate [4], making it a valuable target for studies in evolution and mitochondrial-related diseases. Recent studies using integrated multi-omics approaches have further revealed how dysregulation in these processes, particularly in diabetic conditions, is linked to microRNA-mediated disruption of antioxidant pathways [5,6].

Bioinformatics tools facilitate the computational analysis of mtDNA to study human evolutionary patterns and to uncover associations between mtDNA variants and complex diseases such as neurodegenerative, cardiovascular disease, and cancer [7]. However, applying these computational tools to specific mitochondrial sequencing data can be challenging, especially for non-professional users. Difficulties include software installation, scripting, and data integration. Moreover, each existing tool is typically built for a specific purpose. For example, MitoZ v3.6 [8] provides a comprehensive toolkit for mitochondrial genome assembly and annotation, particularly useful for biodiversity studies, MToolBox v1.2.1 [9] is a widely established pipeline for reconstructing mitochondrial genomes and quantifying heteroplasmy from high-throughput sequencing data. Furthermore, the Genome Analysis Toolkit (GATK) v4.6.0.0 [10] has established a dedicated mitochondrial pipeline based on the Mutect2 caller, which serves as the industry standard for high-sensitivity variant detection.

These robust tools typically operate as standalone command-line utilities developed using different programming languages such as Python v3.11, R v4.4, and Java v21.0. Different input data formats are also required, which increases overall complexity. For example, while MitoZ utilize de novo assembly, it does not perform clinical variant interpretation; similarly, GATK provides excellent variant calling but lacks downstream phylogenetic or haplogroup classification modules. Consequently, Researchers often build custom pipelines by combining multiple specialized tools. Although the pipelines are effective individually, their fragmentation makes data integration complex. This complexity requires strong command-line skills and manual dependency management. As a result, there is a steep learning curve, especially for clinical researchers. Integrating these tools into a unified framework with Graphical User Interface (GUI) would greatly reduce the complexity of mitochondrial analysis and make it more accessible to the broader research community.

Currently, there is no integrated platform that combines these mtDNA tools with various functions into a single, unified environment. Therefore, a comprehensive tool that simplifies the entire pipeline from assembly and annotation to variant detection would significantly lower technical barriers. To address this gap, we developed MitoGEx, an automated, user-friendly computational pipeline specifically designed for human mtDNA analysis. This software functions as an integrated manager that facilitates the execution of a complete analytical workflow within a single interface, enabling the visualization of mitochondrial variant to enhance the interpretation of genetic data. By assembling essential third-party bioinformatics tools, MitoGEx eliminates the necessity for command-line proficiency and allows researchers to transition from raw sequencing data to downstream results by managing the data flow between disparate tools without manual intervention. This approach allows users to input raw data and receive comprehensive annotation results through a simplified interactive process. Furthermore, MitoGEx ensures the inclusion of current biological insights by providing automated updates for the MitImpact v3.1.3 database [11], which serves as the primary resource for mtDNA annotation in MitoGEx. Upon the selection of a specific variant, MitoGEx also provides a visual summary of the severity through a color-coded interface and facilitates easy access to published literature via an integrated application programming interface (API).

To validate the technical performance and practical utility of the platform, we deployed MitoGEx to analyze a validation cohort of 15 human mitochondrial genomes sequenced at Songklanagarind Hospital. This dataset serves as a proof-of-concept ‘Use Case’ to demonstrate the pipeline’s ability to handle diverse input types (blood and tumor WES), accurately classify haplogroups, and detect variants across a range of heteroplasmy levels. By benchmarking these results against variants and haplogroups concordance, we verify the reliability of our platform for clinical and research applications.

By managing mtDNA analysis and bioinformatics data, MitoGEx not only helps identify mutations linked to mitochondrial diseases but also improves understanding of mitochondrial function and evolution. In the era of big data and advanced bioinformatics, MitoGEx shows that programming skills are no longer necessary to unlock the biological importance of mtDNA. MitoGEx is a comprehensive, user-friendly, open-access tool that allows researchers of all backgrounds to explore mtDNA easily and confidently.

## 2. Materials and Methods

### 2.1. Bioinformatics Pipeline

**Figure 1:**
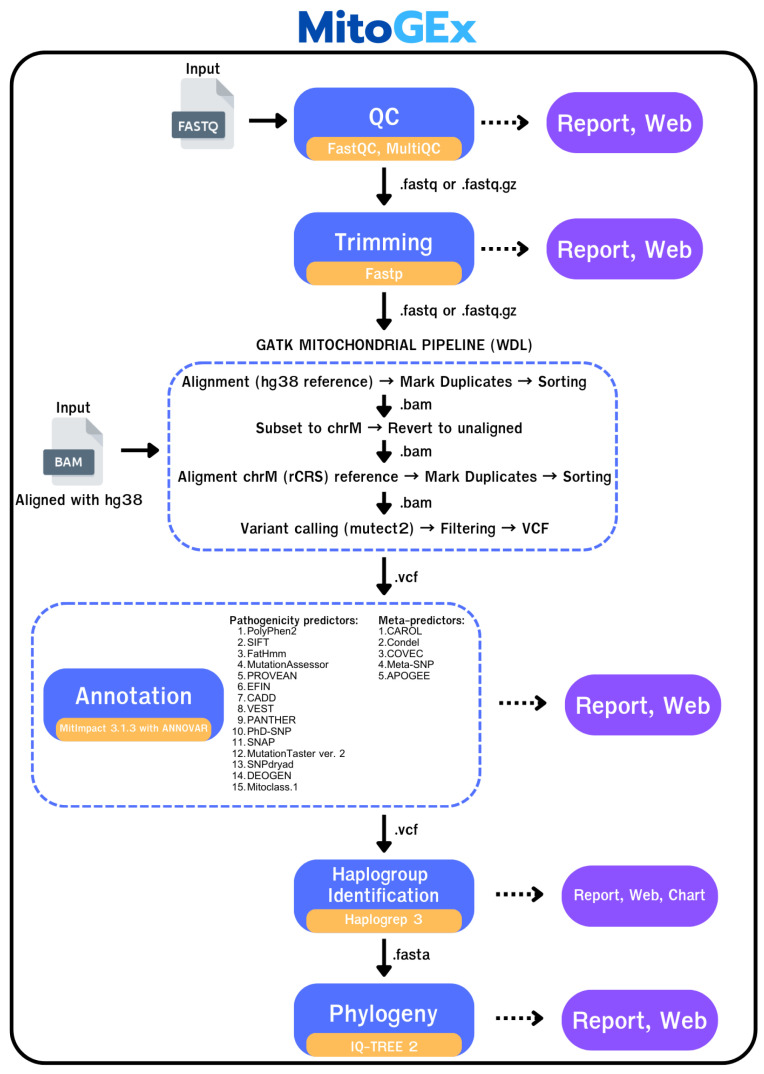
Workflow of the MitoGEx pipeline that combines quality control, alignment, variant calling, annotation, haplogroup identification, and phylogenetic analysis into a single interface. Input files go through FastQC, Fastp, GATK (Mutect2), ANNOVAR v2023Jan05 with MitImpact, Haplogrep 3 v3.2.1, and IQ-TREE 2 v2.0.7, with results automatically summarized in interactive web-based reports and charts. The dotted arrows represent the results from each step of the pipline.

In the first step, 15 raw WES datasets were generated in FASTQ format using the Illumina NextSeq 2000 platform (Illumina, San Diego, CA, USA) with the Agilent SureSelect Human All Exon V6 capture kit (Agilent Technologies, Santa Clara, CA, USA). In the MitoGEx platform process (Figure 1), a FASTQ file was then submitted to the MitoGEx pipeline to initially check its quality using FastQC v0.12.1 [12] or Fastp v1.0.1 [13], which was implemented as part of the pipeline. FastQC was used to assess key quality metrics, including per-base sequence quality, GC content, and sequence duplication levels. Fastp handled quality control, trimming low-quality bases, and removing adapter sequences. The results were compiled into MultiQC 1.32 [14] for the generation of summary reports.

The pre-processed reads were aligned to the human genome (hg38) using BWA v0.7.19 [15], which is integrated into the GATK Mitochondrial Pipeline with workflow description language (WDL). The hg38 reference was selected over older assemblies like hg19 due to its higher performance in characterization of Nuclear Mitochondrial DNA (NUMT) sequences, which is essential for reducing false-positive variant calls during the initial alignment and subsetting phases. This workflow follows the Best Practices developed by Laricchia et al. (2022) [16], which established the Mutect2 --mitochondria-mode based pipeline as the standard for high-sensitivity mitochondrial variant calling. The alignment was performed using the standard command structure bwa mem -K 100000000 -p -v 3 -t [threads] -Y, where the -Y flag is crucial for using soft-clipping for supplementary alignments and -K ensures deterministic output independent of the number of threads.

The resulting alignments were stored in BAM format, with duplicate reads marked, and the alignments were sorted. Mitochondrial reads were then extracted from the BAM files and reverted to an unaligned state before being re-aligned to the mitochondrial genome (chrM) using the revised Cambridge Reference Sequence (rCRS) [17]. Final variant calling was conducted by analyzing the latest alignments with GATK Mutect2 via the MitochondriaPipeline.wdl workflow. To minimize false positives arising from Nuclear Mitochondrial DNA (NUMTs), we applied a multi-stage filtering strategy. First, reads are aligned to the complete hg38 reference genome, allowing nuclear-encoded mitochondrial sequences to map to their homologous nuclear loci rather than the mitochondrial genome. Second, we applied strict read-pair filters during the BAM subsetting phase, specifically the MateOnSameContigOrNoMappedMateReadFilter in Mutect2, to exclude read pairs where one mate anchors to a nuclear chromosome. This approach is particularly vital for the WES data used in this study, where off-target nuclear capture is common.

In the variants calling step, variants were called using --mitochondria-mode, which adjusts internal parameters for circular genomes and high depth. Then, to handle extreme coverage depth typical of mitochondrial sequencing, downsampling was applied using --max-reads-per-alignment-start 75. Heteroplasmy is reported in the final variant call format (VCF) as the Allele Fraction (AF). Variant filtering and post-filtering process were executed using GATK FilterMutectCalls and VariantFiltration, respectively. The filtering step incorporated dynamic contamination estimates calculated by HaploCheck to filter variants that were likely arising from sample contamination. Additionally, a “blacklisted sites” mask, derived from the GATK Resource bundle was applied to exclude regions known for high false-positive rates due to mapping artifacts.

The exact parameters and filters applied are shown in Table 1. The final variants were subsequently annotated for pathogenicity assessment using the integrated MitImpact database in conjunction with the ANNOVAR tool [18]. Variant interpretation incorporated multiple pathogenicity prediction algorithms, including PolyPhen-2 [19,20], SIFT [21], SIFT4G [22], FatHMM [23], MutationAssessor [24], PROVEAN [25], EFIN [26], CADD [27], VEST [28], PANTHER [29,30], PhD-SNP [31], SNAP [32], MutationTaster [33], DEOGEN2 [34], SNPDryad [35], AlphaMissense [36], MLC [37], and Mitoclass.1 [38]. In addition, a series of meta-predictors namely CAROL [39], Condel [40], COVEC [41], Meta-SNP [42], and APOGEE [43,44], and CHASM [45] for cancer-specific prediction were employed to enhance the robustness of the predictive analyses.

#### Benchmarking Strategy

To evaluate the analytical performance of MitoGEx, we utilized a simulated truth dataset from Battle et al. (2022) [46]. This dataset consists of 30 synthetic samples (2000× coverage) with pre-defined mitochondrial variants. The authors also employed Mutect2 for variant calling in their study. This truth dataset allowed for the precise calculation of sensitivity (recall), precision, and false-discovery rates.

### 2.2. Downstream Analysis

A mitochondrial haplogroup is a group of similar mtDNA sequences that share a common ancestor and are characterized by specific sets of genetic variants [47]. These haplogroups provide information about maternal lineage and population history, and they are commonly used in genetics, anthropology, and forensic research [48]. The haplogroup results from this study were reviewed and visualized using the MitoGEx website, which features a graphical interface, producing reports that included haplogroup assignments for individual samples, haplogroup charts, and their corresponding population frequencies.

Mitochondrial haplogroups in MitoGEx were identified using integrated Haplogrep 3 [49], a tool designed to classify mtDNA variants into predefined haplogroups. The process involved comparing the mitochondrial variants detected in each sample with data from PhyloTree 17—Forensic Update 1.2 [50,51] for mtDNA phylogenies. Here, we analyze haplogroups across 15 samples. In HaploGrep 3, haplogroup assignment is carried out using a hierarchical prediction framework. The analysis starts with a broad classification into major haplogroups, which is then refined by identifying lineage-defining mutations. This step-by-step approach enables progressively more specific resolution, allowing for the accurate delineation of mitochondrial lineages from global haplogroups to more narrowly defined sub-haplogroups.

To evaluate the evolutionary relationships among the mitochondrial genomes, a phylogenetic tree was built using IQ-TREE 2 [52]. The input for the analysis consisted of whole mitochondrial genome consensus sequences generated by applying the filtered variants from each sample to the rCRS reference genome. This approach preserves invariant sites, enabling accurate Maximum Likelihood estimation of branch lengths and evolutionary rates. The optimal substitution model was automatically selected using ModelFinder (-m MFP), and branch support was assessed using 1000 replicates was employed to assess the reliability of the tree branches and provide statistical support for the phylogenetic relationships. The resulting phylogenetic tree was visualized with the Phylocanvas software v2016 [53], which automatically generates figures that are responsive and easy to use without customization. Additionally, the Python Environment for Tree Exploration (ETE) Toolkit v3.1.3 [54] was used for further annotation and refined visualization. The ETE Toolkit library enabled the integration of annotations, adjustment of scaling, and customization of visual features to enhance clarity and presentation.

### 2.3. Output and Visualization

The results from Section 2.1 and Section 2.2, including quality control, alignment quality, variant annotation details, haplogroup assignment, and phylogenetic tree analyses, have been compiled into an HTML report. All results were then displayed within an interactive web interface, allowing users to visualize and review the data. Interactive charts, including pie charts that display haplogroups, variant annotation tables, and phylogenetic tree visualizations, were created for easy data exploration. All output files were stored and organized on a designated personal computer. Additionally, these results can be easily shared online, providing a platform for collaboration and sharing findings with other researchers or the broader scientific community.

### 2.4. System Requirements

MitoGEx was developed by combining multiple programming languages, including Java for user interface applications based on Linux systems, Shell scripts for back-end analysis, Python for phylogenetic analysis, and several web programming languages (HTML, PHP, JavaScript) for creating reports with table data and charts. MitoGEx is designed to support whole-exome sequencing data generated from paired-end reads in either FastQ or BAM formats. The platform accommodates both single- and multi-genome analyses within a single workflow. It automates the entire analytical pipeline, including pre-processing, read trimming, reference genome alignment, variant calling, functional annotation, mitochondrial haplogroup assignment, phylogenetic reconstruction, and data visualization.

To ensure ease of installation and scientific reproducibility, MitoGEx provides two primary deployment methods. First, users can utilize a Conda environment v26.1.1 [55] to manage software dependencies, with pre-configured environment files available in the project’s GitHub repository (github.com/mitogex) (accessed on 15 March 2026). Second, MitoGEx now supports containerized deployment via Docker. The Docker image encapsulates the entire computational environment, including all third-party tools and specific library versions, allowing researchers to execute the pipeline across various operating systems without manual dependency configuration. This containerized approach ensures that the analytical environment remains consistent and reproducible regardless of the host system’s architecture. MitoGEx was tested on Ubuntu 22.04 with a CPU with 4 cores and 8 threads, 8 GB of RAM, 15 GB of disk storage, and internet access for database updates and journal publication links from NCBI. The specific versions of all third-party tools integrated into the pipeline such as GATK, Fastp, and HaploGrep 3 are listed in Table 2.

For long-term reproducibility, the complete source code, including WDL scripts and Conda environment files, has been archived on Zenodo (DOI: 10.5281/zenodo.18908614). The pre-configured Docker image is available on Docker Hub (kongpopjeen/mitogex:latest (https://hub.docker.com/r/kongpopjeen/mitogex, accessed on 15 March 2026)), providing a persistent and version-controlled execution environment.

### 2.5. Pipeline Configuration

**Figure 2:**
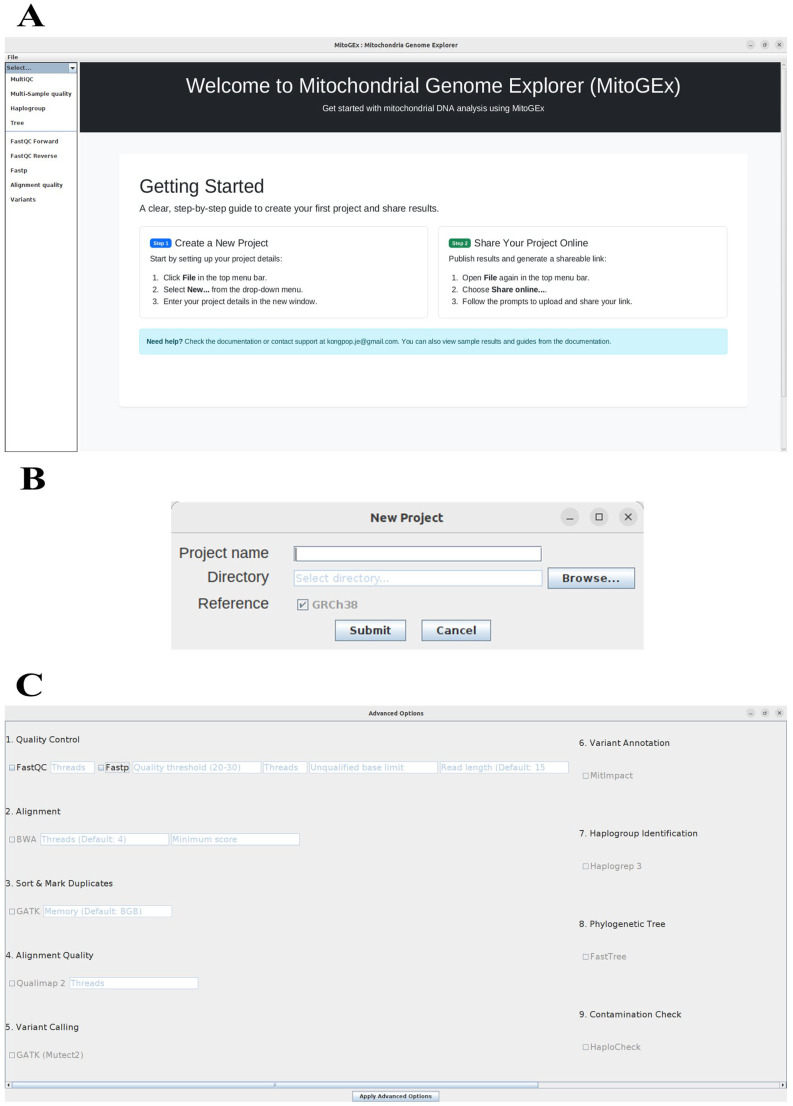
Graphical user interface of the MitoGEx platform for setting up analysis, showing (**A**) the welcome screen; (**B**) project setup dialog; and (**C**) advanced parameter options.

MitoGEx has two modes: quick and advanced, designed for non-professional and experienced users. In quick mode (Figure 1), all processes can be initiated with default parameters using just one click. All tools in this mode are pre-configured for ease of use and simplicity. In advanced mode (Figure 2C), experienced users have the flexibility to manually adjust various parameters, enabling more detailed customization. Users can modify settings for each step of the pipeline, including quality control, alignment, variant calling, and annotation. Essential tools in this mode, such as FastQC, BWA, GATK, and others, are accessible through the graphical user interface (GUI), where users can directly set parameters for threads, memory, read lengths, and quality thresholds. This mode provides precise control over the analysis, allowing advanced users to tailor the workflow to their specific research needs.

### 2.6. Result Sharing and Web-Based Visualization

To bridge the gap between local analysis and web-based visualization, MitoGEx includes an optional result-sharing mechanism that allows users to transfer analysis outputs generated by the GUI-based pipeline to a web-accessible environment. Upon completion of an analysis, MitoGEx produces structured HTML reports containing quality control summaries, haplogroup classification results, variant annotations, and phylogenetic visualizations. These reports are generated locally and remain fully accessible as static files for offline use.

In addition to local storage, MitoGEx provides an optional “Share Results” function. Importantly, the primary MitoGEx analytical engine is a local-only framework, all raw sequencing data and clinical metadata remain on the users local hardware and are never automatically transmitted to external servers. When the sharing option is manually enabled by the user, selected HTML result files are securely transmitted to a web server via an HTTPS-encrypted (TLS/SSL) multipart upload protocol. The communication is managed by a Java-based uploader module that establishes a secure connection to the MitoGEx web server and transfers result files alongside project identifiers and session-specific metadata.

On the server side, a PHP-based receiver script ensures the security of the shared environment by validating token-based authentication, sanitizing user inputs, and enforcing strict file-type restrictions (extension whitelisting) to prevent unauthorized script execution or directory traversal. Uploaded files are organized into non-indexed directories protected by unique, randomized 8-character session IDs to prevent accidental URL discovery and file name collisions. Shared results are retained on the https://mitogex.com/. demo server for a period of 90 days for collaborative review before automatic purging. Users are strongly advised to de-identify all samples and avoid uploading sensitive personally identifiable information (PII) to ensure compliance with data privacy regulations. Once shared, each project is assigned a unique, shareable URL, which is returned to the GUI, allowing users to view interactive results remotely or share analysis outputs with collaborators without requiring local installation of the pipeline.

Importantly, the web application demonstrated in the case studies serves as a reference implementation of this result-sharing capability rather than a mandatory component of the MitoGEx workflow. All analyzed results including quality control, variant detection, haplogroup assignment, and phylogenetic reconstruction are performed entirely within the local GUI pipeline. The web interface does not perform additional computations but instead provides an optional visualization and dissemination layer for results generated by MitoGEx. While the public deployment hosted at https://mitogex.com/ currently showcases example datasets processed by the authors, the same upload mechanism is integrated into the distributed GUI application, allowing any user to deploy a similar web endpoint or use the provided server-side scripts to host and visualize their own results.

This modular architecture ensures that MitoGEx remains fully functional as a standalone desktop pipeline while offering extensibility for web-based result sharing and collaborative analysis.

## 3. Results

### 3.1. Graphical User Interface (GUI)

The MitoGEx pipeline was created as a JAVA-based application that enables users to perform mtDNA analysis through an intuitive graphical user interface (GUI), as shown in Figure 2. To start analysis with MitoGEx, users simply select the project folder containing the input files and run the workflow via the interface, without needing complex command-line steps (Figure 2B). After a one-time installation, all future analyses can be done entirely within the GUI. The pipeline supports both single-sample and multi-sample analysis by specifying the full path to the input folder. All input files must be in standard sequencing formats like FASTQ or BAM and should follow a consistent naming convention (e.g., sample_1.fastq.gz, sample_2.fastq.gz, or sample.bam) to ensure proper recognition and processing by the system.

### 3.2. Technical Validation Case Study: Whole-Exome Sequencing of Human mtDNA

A demonstrated pipeline was used to analyze mtDNA sequences from 15 in-house samples collected from Songklanagarind Hospital, as shown in Table 3.

The cohort consisted of 5 blood samples (A01–A05) and 10 tumor tissue samples (A06–A15). All samples were processed identically using the GATK Mutect2 with the --mitochondrial-mode argument. Unlike nuclear variant calling which relies on a diploid tumor-normal model, this mitochondrial-specific mode is optimized to detect variants across a continuous spectrum of Variant Allele Fractions (VAF), enabling the identification of both heteroplasmy and homoplasmy. For the tumor samples (A06–A15), matched normal (blood) controls were not available for this validation study. Consequently, a direct subtractive analysis to definitively categorize variants as “somatic” (tumor-specific) versus “germline” was not performed during the calling stage. Instead, the output VCFs provide the VAF for every variant, allowing downstream interpretation based on heteroplasmy levels and population frequency. This dataset primarily serves to validate the software’s technical capability to process WES data and report heteroplasmy accurately across different tissue origins.

After quality control, alignment, and variant calling, each sample was classified into a specific mitochondrial haplogroup based on its detected variants. The total number of variants per sample was also recorded. The haplogroups identified across the cohort included M, B, N, F, R, G, and A, with variant counts ranging from 76 to 358 per sample. For example, sample A09, which belongs to haplogroup B4c2, had the highest number of variants (358), while sample A08, classified under haplogroup M17c1, had the lowest (76). Similarly, other samples such as A06 (M13c) and A11 (M7b1a1f) displayed higher variant counts of 305 and 249, respectively. This variation in haplogroup assignments and variant counts reflects the diverse mitochondrial lineages present in this study cohort and provides valuable insights into their genetic composition and evolutionary relationships.

Technical validation using the Battle et al. (2022) [46] gold-standard dataset demonstrated high analytical robustness. Across 30 synthetic samples, MitoGEx identified 2177 true positive variants out of 2270, yielding a precision of 99.32% and a sensitivity of 95.90%. Notably, performance of the MitoGEx remained stable down to a 1% heteroplasmy threshold, confirming its high sensitivity for low-level variants. Furthermore, all 30 samples are in concordance in haplogroup assignment (Supplementary Materials Table S3). These quantitative results establish MitoGEx as a highly accurate platform for comprehensive mitochondrial genome analysis.

A quantitative comparison between a naive (chrM-only) alignment and our competitive mapping pipeline (Supplementary Materials Table S6) revealed that NUMT artifacts accounted for a substantial portion of initial variant calls. Across the 15-sample cohort, the pipeline successfully identified and removed an average of 180 high-quality (PASS) variants that were actually nuclear artifacts.

### 3.3. Quality Control of Sequencing Data

As described in the analysis workflow (Figure 1), quality control was performed as the initial step in analyzing the sequencing data. The results are summarized in Table 4, which shows the output of the MultiQC report generated from each sample processed with Fastp. Each report includes key quality metrics such as the percentage of bases with a Phred score above 30 (% > Q30), the number of reads remaining after filtering, and GC content.

The report indicates that most samples were of high quality, with all exceeding 93% of bases above Q30. For example, sample A02 achieved a Q30 score of 96.2%, while A13 reached 93.67%. GC content values were consistent with expected ranges for mitochondrial genomes, between 48.8% and 56.7%, which are acceptable for mtDNA analysis. Duplication rates remained low, with most samples below 8%. These results confirm that the sequencing data employed for downstream analysis were of adequate quality to ensure reliable variant detection and haplogroup assignment.

### 3.4. Alignment Quality

The alignment quality of all samples was automatically evaluated using Qualimap 2 [56]. The results are summarized in Table 5, which includes mean coverage depth and coverage standard deviation for each sample.

The coverage statistics presented in Table 5 were calculated on a per-base basis across the mitochondrial reference (rCRS). The mean coverage depth across samples varied significantly, ranging from 48.70 × (A10) to 679.24 × (A08), reflecting differences in sequencing depth among samples. Coverage standard deviation showed a similar pattern, with higher variability observed in samples with greater sequencing depth, such as A03 (1776.7) and A01 (1646.9). This high variance relative to the mean is characteristic of Whole-Exome Sequencing (WES) datasets, where mitochondrial reads are captured incidentally (non-specific). This process typically yields highly non-uniform coverage profiles, with extreme depth in specific regions contrasting with lower coverage elsewhere. Despite this non-uniformity, the Mean Mapping Quality remained high across the cohort. These results confirm that the sequencing data were well-aligned, with high mapping accuracy and adequate coverage depth to support downstream analyses, including variant calling and haplogroup classification.

### 3.5. Haplogroup Classification

**Figure 3:**
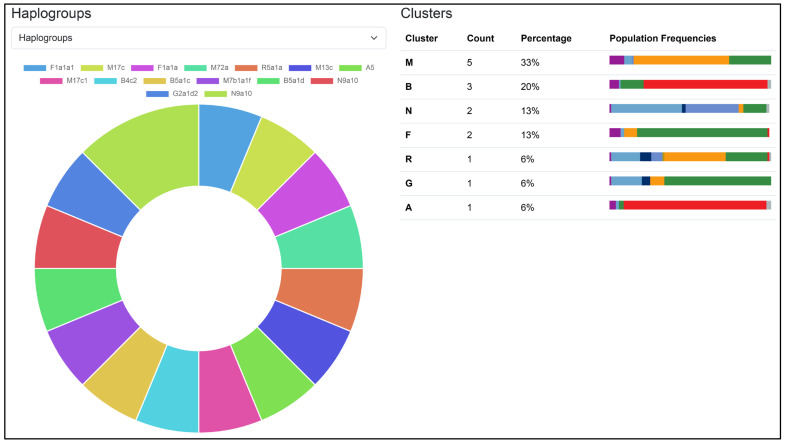
Haplogroup distribution among the 15 samples analyzed. The pie chart shows the relative frequencies of major mitochondrial haplogroups. Cluster M is the most common (33%), followed by clusters B (20%), N (13%), and F (13%), consistent with typical Southeast Asian maternal lineage patterns. The reported percentage may not sum to 100 according to rounding.

The results of haplogroup classification are presented in Figure 3 and Table 6, where Haplogrep 3 software was used to perform the analysis. The results were then collected and displayed in the MitoGEx user interface. The analysis assigned each of the 15 samples to their respective mitochondrial haplogroups based on variant profiles aligned to the PhyloTree 17—Forensic Update 1.2. The samples were distributed across several major haplogroup clusters, with cluster M being the most prevalent (33%), followed by clusters B (20%), N (13%), and F (13%). Clusters R, G, and A were each represented by a single sample (6%), reflecting notable mitochondrial diversity among the analyzed samples. Table 6 provides detailed classification results, including haplogroup assignments, quality scores, coverage values, missing mutations, and private mutations for each sample. The haplogroup quality scores were high across all samples, ranging from 0.826 (A08, M17c1) to 0.974 (A01, F1a1a1), indicating reliable classification. Samples such as A09 (B4c2) and A12 (B5a1d) exhibited a higher number of missing and private mutations. In this context, missing mutations refer to expected haplogroup-defining variants that were not observed in the sample, often due to underrepresentation of certain branches in the reference database. Private mutations are variants present in a sample but absent from PhyloTree 17, representing potential sub-lineage variation or novel mutations not yet captured in the phylogenetic tree.

These findings highlight the genetic heterogeneity of the studied cohort, with the diversity of haplogroup clusters and associated private mutations providing insights into maternal lineage distribution and evolutionary background.

### 3.6. Regional Context from mtDNA Haplogroup

Haplogroup classification was performed using Haplogrep 3, with results summarized in Figure 3. Among the 15 samples, haplogroup assignments covered major maternal lineage clusters: M (33%), B (20%), N (13%), F (13%), along with single representatives of R (6%), G (6%), and A (6%). The most common cluster, M, included sublineages such as M13c, M17c, M17c1, M7b1a1f, and M72a lineages frequently observed in Southeast Asian populations, where haplogroup M can account for up to 60% of maternal ancestry [57,58]. The B cluster, represented by samples B4c2, B5a1c, and B5a1d, is well-known among Southeast Asian and Southern Chinese groups, consistent with its prominence in studies of Thai and Vietnamese populations [59]. Haplogroup F, specifically subclade F1a, was found in samples A01 and A03, matching its wide distribution and high frequency in Thailand and nearby regions [60]. Meanwhile, haplogroup N9a10 is part of the N cluster often found in Tai-Kadai-speaking groups in Thailand and Laos, reflecting northern and northeast Asian maternal migration routes [61]. The less common haplogroups A5, R5a1a, and G2a1d2, each represented by one sample, further indicate maternal lineages with broader East Asian or regional admixture. A and G typically occur at low levels in Mainland Southeast Asia [62], whereas R5 (including R5a1a) is a South Asian marker, indicating South Asian-related ancestry [63]. Haplogroup quality scores ranged from about 0.826 (A08, M17c1) to 0.974 (A01, F1a1a1), confirming the robustness of the classification results. Several samples, including A09 (B4c2) and A12 (B5a1d), showed higher numbers of missing and private mutations suggestive of potential sub-lineage diversity or variants not yet included in PhyloTree. Overall, the haplogroup distribution in this group aligns closely with established patterns of mtDNA diversity in Southeast Asia, with the high prevalence of haplogroups M, B, F, and N matching their documented frequencies in neighboring populations, reinforcing the regional maternal lineage profile seen in previous demographic studies.

### 3.7. Phylogenetic Tree Analysis

**Figure 4:**
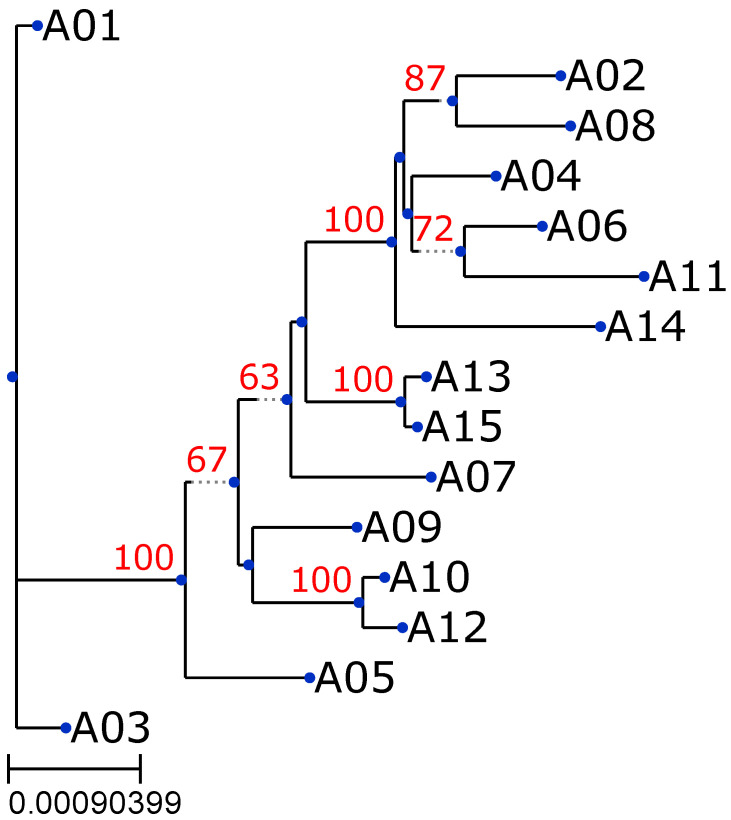
Maximum-likelihood phylogenetic tree of the 15 mitochondrial genomes. The phylogenetic tree was constructed using IQ-TREE 2. While the interactive web interface utilizes Phylocanvas for responsive data exploration, this static figure was generated using the ETE Toolkit to clearly annotate branch support. Bootstrap support values (derived from 1000 replicates) are displayed at the nodes (red text), indicating the statistical confidence of each branch.

To further examine the evolutionary relationships among the mitochondrial genomes, a phylogenetic tree was created using variant data from the mitochondrial genome alignments. The tree was generated with IQ-TREE 2, a robust tool for maximum-likelihood phylogenetic inference. In our MitoGEx, the data were visualized on the website in the Phylocanvas style and using the ETE Toolkit on Java-based software. The resulting tree in Figure 4. Illustrates the relationships among the samples.

The tree reveals a distinct clustering of the samples, reflecting their shared haplogroups and genetic relationships. Sample A01 (F1a1a1) and A02 (M17c) are placed at separate branches, indicating distinct evolutionary paths. Interestingly, samples A13 (N9a10) and A15 (N9a10), both assigned to the same haplogroup, form a closely related cluster, further emphasizing the genetic similarity between these two samples.

Additionally, A06 (M13c) and A11 (M7b1a1f), which belong to different haplogroups, indicate that they are genetically distinct. However, the phylogenetic tree shows that their mitochondrial genomes are relatively similar, as they are positioned close to each other in the tree. These findings suggest that, although the two samples are classified into different haplogroups, they share significant genetic similarities, implying a potentially closer evolutionary relationship than what would be expected based solely on their haplogroup classifications. These phylogenetic results highlight the variability and evolutionary diversity within the dataset, emphasizing the distinct evolutionary paths of the analyzed mitochondrial genomes.

### 3.8. Variant Annotation and Pathogenicity Prediction

When a user clicks the variants tab in the main UI, the Variant Annotation and Pathogenicity Prediction module displays comprehensive functional predictions derived primarily from the MitImpact v3.1.3 database and the ANNOVAR tool. MitoGEx also provides cross-references clinical status reports from the MITOMAP database [64]. MitoGEx displays the original qualitative labels from each individual predictor to maintain the transparency of the results. This approach ensures that the specific scoring definitions of the various algorithms remain intact, allowing researchers to evaluate the data based on their expertise with particular predictors.

**Figure 5:**
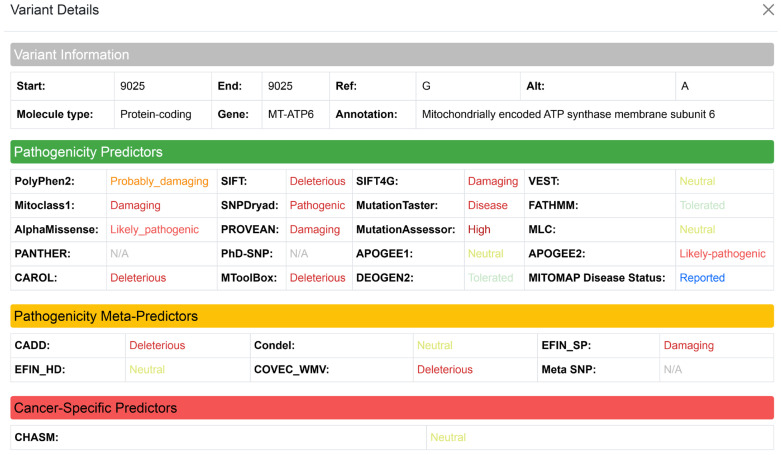
Visualization of *MT-ATP6* 9025 G > A variant annotation in MitoGEx. Screenshot of the MitoGEx interface showing detailed functional annotation and pathogenicity predictions for the *MT-ATP6* 9025 G > A substitution in sample A01. Multiple prediction algorithms classify the variant as deleterious or disease-causing.

An example of this interpretive framework is shown for sample A01 in Figure 5. The identified variant is located at position 9025 (G > A) within the *MT-ATP6* gene, which encodes the mitochondrially encoded ATP synthase membrane subunit 6. This variant elicited mixed predictions, effectively illustrating the platform’s utility in visualizing discordance. While PolyPhen-2 classified the variant as “Probably Damaging” and SIFT/SIFT4G labeled it as “Deleterious” or “Damaging”, additional tools including Mitoclass.1, AlphaMissense, PROVEAN, CAROL, SNPdryad, and MToolBox also predicted a likely pathogenic effect. Furthermore, MutationTaster categorized the variant as “Disease-causing” and MutationAssessor assigned a high functional impact score. Conversely, a subset of predictors, such as VEST, FatHMM, MLC, APOGEE1/2, and DEOGEN2, rated the variant as “Neutral” or “Tolerated,” reflecting inherent variability across prediction algorithms.

In terms of meta-predictors, CADD flagged the variant as deleterious while Condel and EFIN_HD labeled it as neutral. Despite these conflicting computational scores, the MITOMAP database records the status of the variant as “Reported,” which adds essential clinical context from earlier documentation of mitochondrial disorders.

The position 9025 (G > A) occurred within an ATP synthase subunit; the variant may contribute to altered mitochondrial function, supporting the need for further investigation into its potential role in disease mechanisms. This visualization serves as a decision support framework, flagging discordant variants for manual review. Users are encouraged to resolve these conflicts by utilizing the integrated Literature Retrieval API (Section 3.9) to access experimental evidence from PubMed and functional studies, rather than relying solely on computational consensus.

### 3.9. Retriveing Reference Data for Variants in MitoGEx

**Figure 6:**
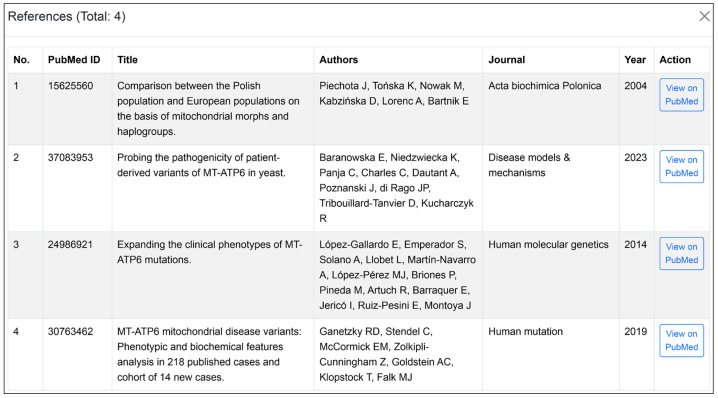
Retrieving reference data for variants using the MitoGEx interface. Demonstration of the automated reference retrieval feature showing the API-driven window that links annotated variants to published studies and clinical databases. Users can access titles, PubMed links, and summary metadata directly within the platform.

In MitoGEx, reference data associated with specific variants, such as the 9025 G > A mutation in the *MT-ATP6* gene, in the previous annotation of the A01 sample, can be easily retrieved through the integrated API feature. This feature allows the platform to automatically access relevant published studies and clinical information linked to the variant. When a user selects a variant, MitoGEx queries the API and displays relevant published studies and literature that mention the variant. Currently, the Literature Retrieval API is powered by NCBI E-utilities (PubMed) through a server-side proxy on https://mitogex.com, which handles secure API key management and rate limiting. This architecture is designed for future extensibility, enabling the server-side integration of additional databases such as ClinVar, PMC, or OMIM, although the current implementation remains focused on PubMed to ensure high-quality, peer-reviewed literature for clinical interpretation. This enables researchers to quickly access relevant information from the scientific literature, facilitating the contextualization of their findings and the exploration of prior research related to the specific mitochondrial mutation. The interface for retrieving these references is illustrated in Figure 6.

## 4. Discussion

The primary contribution of this study is the development of MitoGEx, a user-friendly computational pipeline designed to facilitate broad access to advanced mitochondrial genome analysis. By integrating best-practice bioinformatics tools such as GATK and HaploGrep 3 into a unified graphical interface, the platform addresses the significant technical barriers often associated with command-line bioinformatics. While comparative benchmarking against existing tools such as MitoZ and MToolBox was considered, we identified fundamental methodological discrepancies that preclude a direct, scientifically valid comparison. Specifically, MToolBox relies on the older hg19 reference genome, whereas MitoGEx utilizes the modern hg38 standard. This assembly provides a more comprehensive set of nuclear sequences that function as a decoy for potential NUMT artifacts during the mapping phase. By employing a competitive mapping strategy against the full hg38 nuclear background, MitoGEx effectively captures and filters nuclear-encoded mitochondrial sequences that would otherwise misalign to the mitochondrial genome, ensuring higher specificity for true heteroplasmic calls in WES datasets. Similarly, MitoZ utilizes a de novo assembly approach, which differs inherently from the reference-based alignment method employed by MitoGEx. Therefore, rather than comparing disparate methodologies, we validated MitoGEx against the standard GATK Best Practices pipeline, which has been extensively validated by Laricchia et al. (2022) [16]. Since MitoGEx functions as a GUI wrapper that injects input files directly into the unmodified GATK Workflow Description Language (WDL) scripts, the analytical performance including sensitivity and specificity is intrinsically identical to these established gold standards. Furthermore, we benchmarked the haplogroup classification against the web-based version of HaploGrep 3, achieving 100% concordance across all analyzed samples. Thus, the primary value proposition of MitoGEx lies not in algorithmic novelty, but in significantly reducing technical complexity and setup time while maintaining the rigorous accuracy of standard command-line workflows.

Here, 15 samples of human mitochondrial samples from Songklanagarind Hospital were incorporated for the MitoGEx validation. Notably, the total runtime (Real time) for a single execution of MitoGEx processing the 15 samples (A01–A15) was 13 h, 19 min, and 13 s. When expanded to include 5 additional public samples (A16–A20) for analysis, the total runtime reached 18 h, 34 min, and 5 s, as detailed in Supplementary Materials Table S1. This performance was benchmarked on a Windows host using an Oracle VirtualBox virtual machine (Ubuntu) allocated with 22 CPU cores and 16 GB of RAM. To optimize resource utilization, MitoGEx employs a combination of fixed and dynamic memory allocations: GATK processes are typically allocated 8 GB of RAM by default, while the variant calling stage dynamically adjusts its maximum heap size (Xmx) between 7 GB and 14 GB based on sequencing depth. Furthermore, parallelization is utilized across several stages, including alignment (BWA-MEM2, 4–8 threads) and phylogenetic reconstruction (IQ-TREE 2, auto-threaded). When samples are processed without MitoGEx, users must manually execute each step using the third-party tools listed in Table 2. In such cases, the pause between steps depends on user intervention and may introduce delays or human error. By contrast, this automated framework minimizes the need for manual command line execution, reduces user-related errors, and ensures that sequencing quality metrics are consistently evaluated prior to downstream analyses.

During the initial quality control step, the results demonstrated that the majority of samples exhibited high sequencing quality, with over 92% of bases achieving a Phred score greater than 30. Low duplication rates and consistent GC content further confirmed the reliability of the sequencing data, ensuring that downstream analyses such as variant calling and haplogroup assignment were based on robust datasets. The use of Qualimap 2 demonstrated sufficient coverage depth and high mapping quality across all samples, which is critical for accurate detection of mitochondrial variants and correct haplogroup assignment. MitoGEx can simplify these quality control processes by automatically integrating tools such as FastQC, Fastp, and Qualimap 2 into a single workflow, generating comprehensive summary reports through MultiQC.

In MitoGEx, haplogroup classification of samples is conducted by passing through Haplogrep 3 tools to reveal a diverse distribution of maternal lineages within the cohort. Among the samples, the predominant haplogroups identified were M, B, F, and N, which align with mitochondrial diversity patterns previously reported in Southeast Asian populations. The presence of less common haplogroups, such as A5, R5a1a, and G2a1d2, highlights the heterogeneous maternal ancestry in the study population and suggests historical gene flow and admixture events in the region. Notably, the identification of private mutations in several samples, including A09 (B4c2) and A12 (B5a1d), points to potential sub-lineage diversity that may not yet be fully represented in current reference databases. All classification results were automatically compiled and stored within the pipeline database, alongside the corresponding quality control data.

MitoGEx also provides comprehensive phylogenetic and variant analyses as integral components of its reporting system. The phylogenetic module elucidates evolutionary relationships among mitochondrial genomes, enabling high-resolution visualization of lineage clustering. In the current validation set of 15 samples, sequences were grouped according to their assigned haplogroups, with closely related genomes such as A13 (N9a10) and A15 (N9a10). Furthermore, samples such as A06 (M13c) and A11 (M7b1a1f) exhibited moderate genetic proximity despite belonging to distinct haplogroups. The results demonstrate that while haplogroup classification effectively captures major lineage divisions, phylogenetic reconstruction within MitoGEx offers deeper insights into fine-scale genetic relationships and shared evolutionary history across samples.

The integrated variant analysis module further enhances interpretability by identifying and annotating functionally relevant mutations, including the 9025 G > A substitution in *MT-ATP6*. This variant was annotated using multiple prediction tools and linked to prior publications through an API embedded in MitoGEx. This integration enables users to quickly access reference data and previous studies associated with specific variants, thereby enhancing the biological interpretation of potential pathogenic effects and facilitating hypothesis generation for downstream research. To demonstrate the stability of the tool on external datasets, we additionally processed a subset of five publicly available reference samples from the 1000 Genomes Project using MitoGEx. The results confirm that the platform successfully and consistently processes public reference data. These supplementary validation results, together with the corresponding output reports, are available for review at mitogex.com/shared/samples_1000g_19b03bcf670 (accessed on 15 March 2026). Runtime and performance metrics for each processing step are provided in the Supplementary Materials.

While MitoGEx demonstrated reliable analytical performance, several technical limitations should be acknowledged. First, regarding the specific validation cohort used in this study, the lack of matched normal samples for the tumor tissues prevented the establishment of baseline inherited heteroplasmy. Unlike nuclear DNA, mitochondrial genetics is driven by heteroplasmy levels rather than a strict germline versus somatic classification. Users analyzing cancer datasets are advised that, without matched normals, variant interpretation relies heavily on these heteroplasmy levels and population frequency. To facilitate this, the MitoGEx software automatically extract and explicitly report the exact heteroplasmy level for every detected variant in the final output tables. In studies lacking matched controls, we recommend that users apply a post-analysis filtering threshold of ≥5% heteroplasmy. This conservative threshold empowers researchers to distinguish high-confidence mitochondrial variants from the baseline ‘noise’ of inherited low-level heteroplasmy and sequencing artifacts. Second, regarding operating system compatibility, the application is natively supported on Linux-based systems (Ubuntu), For users on Windows 11, we recommend utilizing the Windows Subsystem for Linux GUI (WSLg), which allows the MitoGEx GUI and its Linux-based backend to be executed natively as a desktop application. This setup significantly lowers the technical barrier for non-professional users by removing the need for a dedicated Linux installation. Alternatively, MitoGEx can be deployed via a pre-configured Docker container, which ensures all bioinformatics dependencies are correctly installed and ready for use on Windows systems.

Overall, MitoGEx serves as a comprehensive and integrative platform for mitochondrial genome analysis, unifying quality control, variant detection, haplogroup classification, phylogenetic reconstruction, and literature-based annotation within a single, user-friendly framework. The analysis of 15 in-house samples demonstrates the robustness and reliability of the MitoGEx pipeline in producing high-quality, interpretable mitochondrial genomic data. The resulting findings are consistent with established population-level diversity and evolutionary patterns, thereby validating the pipeline’s analytical accuracy and its applicability for both research and clinical investigations. By lowering the computational barrier, MitoGEx has the potential to expand the accessibility of mitochondrial research and support investigations into disease-associated variants, population genetics, and evolutionary biology.

## 5. Conclusions

This study introduces MitoGEx as a streamlined solution to the fragmentation of mitochondrial bioinformatics tools. By encapsulating industry-standard algorithms (GATK, HaploGrep 3, IQ-TREE 2) within an accessible graphical interface, the software simplifies complex analyses that typically require multiple standalone tools, effectively removing the programming barrier for clinical and biological researchers. The implementation of both Quick and Advanced modes provides flexibility for users of different expertise levels, while the integrated visualization modules enhance interpretability. The validation on human WES samples demonstrates that MitoGEx delivers analytical accuracy comparable to command-line gold standards while significantly enhancing workflow reproducibility. Overall, MitoGEx (available at mitogex.com and github.com/mitogex) (accessed on 15 March 2026) offers a reproducible and accessible solution for mtDNA analysis, with strong potential for applications in genomics, evolutionary biology, and clinical diagnostics, particularly in settings where bioinformatics support is limited.

## Figures and Tables

**Table 1:** GATK Mitochondrial Pipeline Parameters and Filtering Thresholds used in MitoGEx.

Step	Parameter/Filter	Command	Description
Preprocessing	Alignment Strategy	BWA-MEM -K 100000000 -p -v 3 -t 2 -Y	Alignment to rCRS with soft-clipping of supplementary alignments.
NUMT Control	Read Filters	MateOnSameContigOrNoMappedMateReadFilter	Discards reads where the mate pairs to a nuclear chromosome.
		MateUnmappedAndUnmappedReadFilter	Discards pairs where both reads are unmapped.
Variant Calling	Tool	Mutect2 --mitochondria-mode	Sets mitochondrial-specific likelihoods and priors.
	Downsampling	--max-reads-per-alignment-start 75	Caps coverage to prevent system saturation.
	MNP Handling	--max-mnp-distance 0	Disables merging of nearby variants into MNPs.
	Annotations	StrandBiasBySample	Annotates strand bias for filtering.
Filtering	Tool	FilterMutectCalls	Main filtering engine.
	Contamination	--contamination-estimate [Value]	Dynamic value derived from HaploCheck per sample.
	Allele Count	--max-alt-allele-count 4	Restricts the maximum number of alt alleles at a locus.
	F-Score Beta	--f-score-beta 1.0	Balances recall and precision (default setting).
Post-Filtering	Masking	VariantFiltration—mask [BED]	Hard filters variants falling in problematic “blacklisted” regions.

**Table 2:** Third-party tools, versions, and functions integrated into MitoGEx.

Tool	Version	Function
FastQC	0.12.1	Quality control metrics and assessment
Fastp	1.0.1	Quality control, read trimming, and adapter removal
MultiQC	1.32	Aggregation of quality control reports
GATK	4.6.0.0	Mitochondrial variant calling pipeline
Qualimap 2	2.3	Alignment quality assessment
Haplogrep 3	3.2.1	Mitochondrial haplogroup classification
IQ-TREE 2	2.0.7	Phylogenetic tree reconstruction
BWA	0.7.19	Sequence alignment to reference genome
Samtools	1.22.1	BAM/SAM file manipulation and indexing
ETE Toolkit	3.1.3	Phylogenetic tree visualization and annotation
Cromwell	87	WDL workflow execution engine

**Table 3:** Summary of MitoGEx analysis results for 15 samples from Songklanagarind Hospital. Samples A01–A05 were isolated from blood, and samples A06–A15 came from tumor tissue.

Sample Name	Haplogroup	Total Variants
A01	F1a1a1	258
A02	M17c	91
A03	F1a1a	90
A04	M72a	121
A05	R5a1a	79
A06	M13c	305
A07	A5	164
A08	M17c1	76
A09	B4c2	358
A10	B5a1c	182
A11	M7b1a1f	249
A12	B5a1d	165
A13	N9a10	212
A14	G2a1d2	241
A15	N9a10	229

**Table 4:** MultiQC summary of quality control statistics for 15 blood and tissue mtDNA samples. Each row shows an individual sample processed with Fastp. The table includes duplication percentage, proportion of bases > Q30, total reads retained after filtering, and GC content.

Sample	% Duplication	% > Q30	Million Reads After Filtering	%GC Content
A01	11.54	94.19	128.20	49.50
A02	6.02	96.20	79.40	48.84
A03	6.31	96.13	79.03	49.06
A04	6.54	96.21	73.99	48.99
A05	5.65	96.12	72.24	48.91
A06	7.44	94.88	72.98	54.74
A07	6.94	94.84	75.30	52.53
A08	6.58	94.76	74.65	52.11
A09	4.47	94.14	72.85	56.68
A10	6.82	94.62	69.12	52.85
A11	3.99	93.97	68.78	54.15
A12	9.14	94.47	73.50	51.77
A13	8.09	93.67	73.91	51.21
A14	7.08	94.85	68.95	53.36
A15	5.74	94.60	64.89	52.75

**Table 5:** Alignment statistics of mitochondrial reads after mapping to the rCRS reference. The table shows mean coverage depth, standard deviation, mean mapping quality for each sample, and total mapped reads, confirming the accuracy and uniformity of alignments produced by the GATK mitochondrial pipeline.

Sample	Coverage Mean (×)	Coverage Std (×)	Mean Mapping Quality	Total Mapped Reads
A01	384.29	1646.91	59.98	42,551
A02	228.05	1081.37	59.99	25,587
A03	404.55	1776.66	59.99	45,315
A04	294.57	1354.96	59.99	32,915
A05	208.39	948.53	59.99	23,290
A06	117.30	50.64	59.99	13,370
A07	62.05	27.34	59.99	7076
A08	679.24	209.34	59.99	76,574
A09	112.28	41.90	59.98	12,756
A10	48.70	21.19	59.96	5514
A11	52.72	20.45	59.99	6011
A12	192.86	69.65	59.97	22,000
A13	219.93	61.56	59.99	24,751
A14	54.96	22.02	59.99	6242
A15	99.31	35.43	59.99	11,251

**Table 6:** Haplogroups classification results generated by HaploGrep 3. Each sample is listed with its assigned haplogroup, classification quality score, coverage, number of missing mutations, and global private mutations according to PhyloTree 17—Forensic Update 1.2.

Sample Name	Haplogroup	Quality	Coverage	Missing Mutations	Global Private Mutations
A01	F1a1a1	0.9749	16,569	249d	247G 12346T
A02	M17c	0.9114	16,569		290A 310T 709A 1842G 2746C 4529G 10319G 11914A 14470C 14544A 15517T 16189C
A03	F1a1a	0.9469	16,569	249d	247G 310T 513G 12501C 15299C 15340G 15643T 16311C
A04	M72a	0.9516	16,569	16166d	310T 11151T 13615G 15932C 16161T
A05	R5a1a	0.9506	16,569	200G	310T 513G 5580C 16166G 16311C
A06	M13c	0.9306	16,569	152C 6023A 16381C	150T 310T 3882A 9258T 10907C
A07	A5	0.9342	16,569		310T 513G 1709A 16126C 16234T 16265G 16445C
A08	M17c1	0.826	16,569	143A 2706G 8860G	259G 310T 513G 9377G 11150A 14845T 16111A 16140C 16311C 16352C 16353T
A09	B4c2	0.8492	16,569	8281d 8282d 8283d 8284d 8285d 8286d 8287d 8288d 8289d 8860G 16184A	310T 8270C 16182A 16311C
A10	B5a1c	0.878	16,569	4769G 8281d 8282d 8283d 8284d 8285d 8286d 8287d 8288d 8289d	152C 310T 513G 8270C
A11	M7b1a1f	0.9254	16,569	263G 4048A 8860G 16192T 16297C	310T 332T 16189C 16296C 16298C
A12	B5a1d	0.9065	16,569	8281d 8282d 8283d 8284d 8285d 8286d 8287d 8288d 8289d	513G 8270C 14756G
A13	N9a10	0.8859	16,569	5417A 8860G	1027G 5554T 6956C 16189C
A14	G2a1d2	0.929	16,569	14766T 16227G	310T 8964T 15238T 15930A 16150T 16192T
A15	N9a10	0.9255	16,569		

## Data Availability

The human sequencing data analyzed in this study are protected under the Ethics Committee of Prince of Songkla University and are not publicly available. Qualified researchers may request access to de-identified datasets by contacting the corresponding author and submitting a formal research proposal to the Ethics Committee. For technical validation and benchmarking, we utilized publicly available datasets from Battle et al. (2022) [46] and the 1000 Genomes Project (Sample IDs: HG00152, HG01028, NA18531, NA18636, and NA19084). The complete execution log files for all validation samples (A01–A20) are provided via the links in Supplementary Materials Table S1.

## Supplementary Materials

The following supporting information can be downloaded at: https://www.mdpi.com/article/10.3390/genes17030338/s1, Table S1. Summary of runtime for each step implemented in the MitoGEx workflow. Table S2. Overall performance summary of MitoGEx variant calling against benchmark datasets. Table S3. Performance metrics of MitoGEx on a per-sample basis for the benchmark dataset. Table S4. Detailed list of false positive and false negative variants identified by MitoGEx. Table S5. Detection limits of heteroplasmy thresholds on MitoGEx detection performance on benchmark datasets. Table S6. Quantitative Impact of NUMT Filtering. A naive pipeline (mapping only to chrM) against our MitoGEx pipeline (competitive mapping to hg38). Across 15 samples (A01–A15).

## Abbreviations

The following abbreviations are used in this manuscript:

VCF	Variant Call Format
GATK	Genome Analysis Toolkit
BWA	Burrows Wheeler Aligner
BAM	Binary Alignment Map
WDL	Workflow Description Language
ANNOVAR	ANNOtate VARiation
HTML	Hypertext Markup Language
API	Application Programming Interface
