# Supplementary material for: MitoGEx: An Integrated Platform for Streamlined Human Mitochondrial Genome Analysis
Source: Genes (Basel). 2026 Mar 18;17(3):338. doi: 10.3390/genes17030338 (PMC13026451; doi:10.3390/genes17030338)
Supplement: Supplementary file 1 [file genes-17-00338-s001.zip › genes-4166153-supplementary.pdf]

Supporting Information

MitoGEx: An Integrated Platform for Streamlined Human Mitochondrial Genome Analysis

Kongpop Jeenkeawpiam [1,3], Pemikar Srifa [1,3,*], Natakorn Nokchan [3], Natthapon Khongcharoen [1,3], Anas Binkasem [1,3] and Surasak Sangkhathat [2,3,*]

1 Department of Biomedical Sciences and Biomedical Engineering, Faculty of Medicine, Prince of Songkla University, Songkhla, Thailand 90110;
2 Department of Surgery, Faculty of Medicine Siriraj Hospital, Mahidol University, Bangkok, Thailand 10700;
3 Translational Medicine Research Center, Faculty of Medicine, Prince of Songkla University, Songkhla, Thailand 90110;
* Correspondence: pemikar.s@gmail.com, surasak.sag@mahidol.ac.th

Table S1. Summary of runtime for each step implemented in the MitoGEx workflow. Data from Samples A01 to A05 were selected to demonstrate the runtime performance of the validation sample set described in the main manuscript. Data from Samples A16 to A20 represent examples of analyses performed on publicly available reference samples from the 1000 Genomes Project using MitoGEx (See results in **https://mitogex.com/shared/samples_1000g_19b03bcf670**). All information was extracted from the MitoGEx log file generated during a single run of all 20 samples (A01-A20), which is provided in https://mitogex.com/log/MitoGEx.log.A01_A20.txt.

<table>
<tr><th rowspan="2">Sample</th><th rowspan="2">Quality control, read trimming, and adapter removal using Fastp (second)</th><th colspan="2">Sequence alignment to reference genome with BWA (hg38)</th><th rowspan="2">Sort & mark duplicates (mm:ss)</th><th rowspan="2">Mitochondrial Pipeline for Variant calling with GATK (mm:ss)</th><th rowspan="2">Alignment quality assessment with Qualimap 2 (second)</th><th rowspan="2">Haplogroup classification By Haplogrep 3 (second)</th><th rowspan="2">Phylogenetic tree reconstruction using IQ-TREE 2 (second)</th></tr>
<tr><th>Real time (second)</th><th>CPU time (second)</th></tr>
<tr><td>A01</td><td>817</td><td>2682.485</td><td>21037.451</td><td>25:46</td><td rowspan="5">~ 4:30</td><td>3</td><td rowspan="10">23</td><td rowspan="10">69</td></tr>
<tr><td>A02</td><td>304</td><td>1656.247</td><td>12789.416</td><td>10:93</td><td>1</td></tr>
<tr><td>A03</td><td>322</td><td>1648.410</td><td>12639.753</td><td>11:59</td><td>1</td></tr>
<tr><td>A04</td><td>295</td><td>1610.567</td><td>12442.154</td><td>10:84</td><td>2</td></tr>
<tr><td>A05</td><td>294</td><td>1536.597</td><td>11789.228</td><td>10:25</td><td>1</td></tr>
<tr><td>A16</td><td>359</td><td>869.274</td><td>6000.243</td><td>9:44</td><td rowspan="5">~ 2:50 to 3:20</td><td>1</td></tr>
<tr><td>A17</td><td>232</td><td>584.037</td><td>4130.332</td><td>5:40</td><td>1</td></tr>
<tr><td>A18</td><td>326</td><td>759.528</td><td>5329.950</td><td>8:39</td><td>1</td></tr>
<tr><td>A19</td><td>756</td><td>1831.354</td><td>14166.975</td><td>25:04</td><td>2</td></tr>
<tr><td>A20</td><td>202</td><td>674.401</td><td>4891.458</td><td>5:37</td><td>1</td></tr>
<tr><td>Overall 20 samples</td><td colspan="8">18:34:05 (hh:mm:ss)</td></tr>
</table>

Note: Runtimes are reported as wall-clock time (Real time). Hardware: Oracle VirtualBox VM (22 cores, 16 GB RAM). In the absence of real-time memory profiling logs, we report the maximum memory allocation (Xmx) where explicitly defined in the software: Picard (8 GB) and Mutect2 (7–14 GB depending on coverage). Other stages utilize dynamic system-level memory allocation (JVM defaults). All pipeline stages successfully executed within the 16 GB system RAM.

The MitoGEx log file generated during a single run of all 15 validation samples (A01-A15) included in the manuscript is available at:
https://mitogex.com/log/MitoGEx.log.A01_A15.txt

The MitoGEx log file generated during a single run of all 5 reference samples (A15-A20) from the 1000 Genomes Project is available at:
https://mitogex.com/log/MitoGEx.log.A16-A20.txt

Table S2. Overall performance summary of MitoGEx variant calling against benchmark datasets.
This table summarizes the aggregate performance of MitoGEx across all 30 benchmark samples. It presents the total counts of True Positives (TP), False Positives (FP), and False Negatives (FN), along with the overall Precision, Recall, and F1-score, providing a high-level evaluation of the MitoGEx diagnostic accuracy.

Tool	TP	FP	FN	Precision	Recall	F1
MitoGEx	2177	15	93	0.993157	0.959031	0.975796

Table S3. Performance metrics of MitoGEx on a per-sample basis for the benchmark dataset.

This table provides a detailed breakdown of the benchmarking results for each of the 30 individual samples, including identified haplogroups, counts of True Positives (TP), False Positives (FP), and False Negatives (FN), Total variants of truth datasets, Precision, Recall, and F1-scores are calculated for each sample relative to the gold-standard truth dataset.

Sample	Haplogroups	TP	FP	FN	Variants of truth datasets	Precision	Recall	F1
sample_A	A2+(64)	70	2	6	76	0.972222	0.921053	0.945946
sample_B	B2	68	2	3	71	0.971429	0.957746	0.964539
sample_C	C	79	3	6	85	0.963415	0.929412	0.946108
sample_D	D4	71	0	2	73	1	0.972603	0.986111
sample_E	E1a1a1	78	0	3	81	1	0.962963	0.981132
sample_F	F1	65	1	4	69	0.984848	0.942029	0.962963
sample_G	G2a'c	73	0	2	75	1	0.973333	0.986486
sample_H	H	50	0	2	52	1	0.961538	0.980392
sample_HV	HV	51	0	3	54	1	0.944444	0.971429
sample_I	I	72	0	2	74	1	0.972973	0.986301
sample_J	J1c	69	0	2	71	1	0.971831	0.985714
sample_K	K1a	71	0	2	73	1	0.972603	0.986111
sample_L0	L0a1	118	3	7	125	0.975207	0.944000	0.959350
sample_L1	L1b	108	1	4	112	0.990826	0.964286	0.977376
sample_L2	L2a1	90	0	2	92	1	0.978261	0.989011
sample_L3	L3	63	0	2	65	1	0.969231	0.984375
sample_L4	L4b1a	90	0	2	92	1	0.978261	0.989011
sample_L5	L5a	112	2	7	119	0.982456	0.941176	0.961373
sample_M	M	66	0	2	68	1	0.970588	0.985075
sample_N	N1b1	73	0	3	76	1	0.960526	0.979866
sample_P	P9a	65	1	5	70	0.984848	0.928571	0.955882

sample_R	R	55	0	2	57	1	0.964912	0.982143
sample_S	S3	45	0	2	47	1	0.957447	0.978261
sample_T	T2	72	0	3	75	1	0.960000	0.979592
sample_U	U5a'b	60	0	2	62	1	0.967742	0.983607
sample_V	V	55	0	3	58	1	0.948276	0.973451
sample_W	W	73	0	3	76	1	0.960526	0.979866
sample_X	X2b+226	72	0	2	74	1	0.972973	0.986301
sample_Y	Y1	65	0	3	68	1	0.955882	0.977444
sample_Z	Z3	78	0	2	80	1	0.975000	0.987342

Table S4. Detailed list of false positive and false negative variants identified by MitoGEx.

This table catalogs every discordant variant identified during the benchmarking process. Each entry specifies the sample ID, error type (False Positive or False Negative), genomic position on the mitochondrial reference, variant type (SNP, Insertion, or Deletion), and the specific nucleotide details.

Sample	Type	Pos	VarType	Detail
sample_A	False Positive	513	DEL	GCA
	False Positive	16318	DEL	AG
	False Negative	522	DEL	CA
	False Negative	310	INS	C
	False Negative	3107	DEL	NT
	False Negative	16315	DEL	TA
	False Negative	308	INS	C
	False Negative	521	DEL	AC
sample_B	False Positive	8270	DEL	CACCCCCTCT
	False Positive	16358	SNP	G
	False Negative	3107	DEL	NT
	False Negative	314	INS	C
	False Negative	8279	DEL	TACCCCCTCT
	False Positive	513	DEL	GCA
	False Positive	512	SNP	T

sample_C	False Positive	285	DEL	CAA
	False Negative	288	DEL	AAA
	False Negative	522	DEL	CA
	False Negative	3107	DEL	NT
	False Negative	314	INS	C
	False Negative	514	SNP	T
	False Negative	521	DEL	AC
sample_D	False Negative	3107	DEL	NT
	False Negative	314	INS	C
sample_E	False Negative	3107	DEL	NT
	False Negative	308	INS	C
	False Negative	310	INS	C
	False Positive	513	DEL	GCA
	False Negative	3107	DEL	NT
	False Negative	314	INS	C
	False Negative	521	DEL	AC
	False Negative	522	DEL	CA
sample_G	False Negative	3107	DEL	NT
	False Negative	314	INS	C
sample_H	False Negative	3107	DEL	NT
	False Negative	314	INS	C
sample_HV	False Negative	3107	DEL	NT
	False Negative	308	INS	C
	False Negative	310	INS	C
sample_I	False Negative	3107	DEL	NT
	False Negative	314	INS	C
sample_J	False Negative	3107	DEL	NT
	False Negative	314	INS	C
sample_K	False Negative	3107	DEL	NT
	False Negative	314	INS	C

sample_L0	False Positive	513	DEL	GCA
	False Positive	16189	INS	GC
	False Positive	16183	DEL	ACC
	False Negative	16188	SNP	G
	False Negative	522	DEL	CA
	False Negative	16189	SNP	C
	False Negative	3107	DEL	NT
	False Negative	314	INS	C
	False Negative	16187	SNP	T
	False Negative	521	DEL	AC
sample_L1	False Positive	513	DEL	GCA
	False Negative	3107	DEL	NT
	False Negative	314	INS	C
	False Negative	521	DEL	AC
	False Negative	522	DEL	CA
sample_L2	False Negative	3107	DEL	NT
	False Negative	314	INS	C
sample_L3	False Negative	3107	DEL	NT
	False Negative	314	INS	C
sample_L4	False Negative	3107	DEL	NT
	False Negative	314	INS	C
sample_L5	False Positive	513	DEL	GCA
	False Positive	16189	INS	CC
	False Negative	522	DEL	CA
	False Negative	16189	SNP	C
	False Negative	16179	DEL	CA
	False Negative	3107	DEL	NT
	False Negative	314	INS	C
	False Negative	16187	SNP	T
	False Negative	521	DEL	AC

sample_M	False Negative	3107	DEL	NT
	False Negative	314	INS	C
sample_N	False Negative	3107	DEL	NT
	False Negative	308	INS	C
	False Negative	310	INS	C
sample_P	False Positive	513	DEL	GCA
	False Negative	522	DEL	CA
	False Negative	310	INS	C
	False Negative	3107	DEL	NT
	False Negative	308	INS	C
	False Negative	521	DEL	AC
sample_R	False Negative	3107	DEL	NT
	False Negative	314	INS	C
sample_S	False Negative	3107	DEL	NT
	False Negative	314	INS	C
sample_T	False Negative	3107	DEL	NT
	False Negative	308	INS	C
	False Negative	310	INS	C
sample_U	False Negative	3107	DEL	NT
	False Negative	314	INS	C
sample_V	False Negative	3107	DEL	NT
	False Negative	308	INS	C
	False Negative	310	INS	C
sample_W	False Negative	3107	DEL	NT
	False Negative	308	INS	C
	False Negative	310	INS	C
sample_X	False Negative	3107	DEL	NT
	False Negative	314	INS	C
sample_Y	False Negative	3107	DEL	NT
	False Negative	308	INS	C

	False Negative	310	INS	C
sample_Z	False Negative	3107	DEL	NT
	False Negative	314	INS	C

Table S5. Detection limits of heteroplasmy thresholds on MitoGEx detection performance on benchmark datasets.
This table evaluates the stability of MitoGEx performance across varying heteroplasmy detection thresholds, ranging from 0.01 to 0.1. It summarizes how the change in threshold affects the overall precision and recall of variant calling.

Threshold	Precision	Recall	False Positives
1%	0.993157	0.959031	15
2%	0.993157	0.959031	15
5%	0.993157	0.959031	15
10%	0.993157	0.959031	15

Table S6. Quantitative Impact of NUMT Filtering. A naive pipeline (mapping only to chrM) against our MitoGEx pipeline (competitive mapping to hg38). Across 15 samples (A01–A15).

Sample	Naive_PASS variants	MitoGEx_PASS variants	Shared variants	Artifacts_Removed
A01	405	36	36	369
A02	130	44	42	88
A03	476	40	40	436
A04	517	51	45	472
A05	123	29	29	94
A06	393	45	40	353
A07	35	34	33	2
A08	125	44	43	82
A09	230	49	47	183
A10	41	33	32	9
A11	196	43	43	153
A12	268	49	49	219
A13	54	34	34	20
A14	51	45	45	6
A15	260	35	35	225
